# Receptor for Advanced Glycation End Products and Its Involvement in Inflammatory Diseases

**DOI:** 10.1155/2013/403460

**Published:** 2013-09-11

**Authors:** Yaw Kuang Chuah, Rusliza Basir, Herni Talib, Tung Hing Tie, Norshariza Nordin

**Affiliations:** ^1^Department of Human Anatomy, Faculty of Medicine and Health Sciences, University Putra Malaysia, 43400 Serdang, Selangor, Malaysia; ^2^Department of Pathology, Faculty of Medicine and Health Sciences, University Putra Malaysia, 43400 Serdang, Selangor, Malaysia; ^3^Department of Obstetrics & Gynecology, Faculty of Medicine and Health Sciences, University Putra Malaysia, 43400 Serdang, Selangor, Malaysia

## Abstract

The receptor for advanced glycation end products (RAGE) is a transmembrane receptor of the immunoglobulin superfamily, capable of binding a broad repertoire of ligands. RAGE-ligands interaction induces a series of signal transduction cascades and lead to the activation of transcription factor NF-*κ*B as well as increased expression of cytokines, chemokines, and adhesion molecules. These effects endow RAGE with the role in the signal transduction from pathogen substrates to cell activation during the onset and perpetuation of inflammation. RAGE signaling and downstream pathways have been implicated in a wide spectrum of inflammatory-related pathologic conditions such as arteriosclerosis, Alzheimer's disease, arthritis, acute respiratory failure, and sepsis. Despite the significant progress in other RAGE studies, the functional importance of the receptor in clinical situations and inflammatory diseases still remains to be fully realized. In this review, we will summarize current understandings and lines of evidence on the molecular mechanisms through which RAGE signaling contributes to the pathogenesis of the aforementioned inflammation-associated conditions.

## 1. Introduction

The receptor for advanced glycation end products (RAGE), which belongs to the immunoglobulin superfamily, was first identified and described in terms of its ability to bind advanced glycation end products (AGEs) [1, 2]. This explains the choice of “RAGE” to name this receptor. Due to the ability of RAGE to recognize three-dimensional structures rather than specific amino acid sequences, RAGE is capable of engaging a diverse class of ligands that lack sequence similarities. Because of this feature, this multiligand receptor can therefore be considered a pattern-recognition receptor (PRR) [1, 3]. Ligands that have been found to be recognized by RAGE include AGEs [1], amyloid **β**-peptide [4], DNA binding protein high mobility group box-1 (HMGBl)/amphoterin [5], and S100/calgranulins [6]. In humans and mice, the gene encoding RAGE is located on chromosome 6 close to major histocompatibility complex III (MHC class III), in the vicinity of the genes for lymphotoxin, tumour necrosis factor (TNF), and the homeobox gene *HOX12* [7, 8]. Translation of the mRNA transcribed from this human RAGE gene (~1.4 kb) results in a protein of 404 amino acids with a molecular mass of about 55 kDa [2].

**Figure 1:**
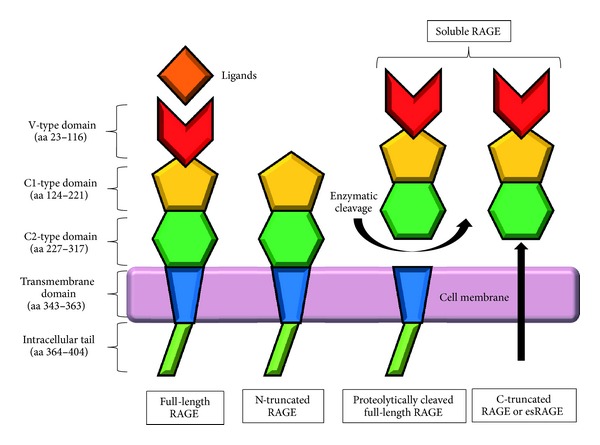
Schematic representation of full-length RAGE and its splice variants. RAGE is composed of an intracellular tail, a transmembrane domain, and an extracellular domain consisting of three immunoglobulin-like domains, one V-type followed by two C-type (C1 and C2) domains. The V-type domain is essential for ligand binding, and deletion of this domain results in an N-truncated form. The C-truncated, circulating soluble RAGE corresponds to the extracellular domain of RAGE lacking the intracellular tail and transmembrane domains. It may derive via proteolytic cleavage of full-length RAGE from the cell surface (cRAGE) or via alternative splicing of RAGE mRNA (esRAGE). C: constant; V: variable.

RAGE is composed of a single hydrophobic transmembrane-spanning domain, a highly charged cytosolic tail, and an extracellular region (Figure 1). This extracellular region consists of one N-terminal V-type immunoglobulin domain and two C-type (C1 and C2) immunoglobulin domains [3, 9]. A flexible linker separates the fully independent C2 domain from the integrated structural unit formed by the V-type and C1 domains [10]. The V-type domain is considered as the principal site for interactions between RAGE and potential extracellular ligands [10, 11].

On the other hand, the short cytosolic tail, which lacks known signalling motifs like kinase domains or phosphorylation sites, has been shown to be critical for RAGE-mediated intracellular signalling [3, 9]. A truncated form of RAGE, in which the cytosolic tail is deleted, has been used to prove the essentiality of this cytosolic tail in intracellular signalling. Although this form of RAGE is capable of recognizing and binding to the RAGE ligands like the wild-type receptor, it cannot mediate any cellular activation upon ligand ligation [6].

## 2. RAGE Expression

RAGE can be constitutively or inducibly expressed in different cells, depending on the cell type and developmental stage [5, 12]. During embryonic development, RAGE is highly expressed in a constitutive manner [12]. Compared to embryonic cells, there is relatively low expression of RAGE in a wide range of differentiated adult cells such as cardiomyocytes, neurons, neutrophils, monocytes/macrophages, lymphocytes, dendritic cells (DCs), and vascular endothelial cells [12, 13].

Unlike constitutive RAGE expression during embryonic development, RAGE is expressed in a regulated manner in adult life. This means that RAGE expression can be induced in situations when there is an accumulation of ligands and inflammatory mediators [3, 14]. However, skin and lung have been identified to be exceptions, as RAGE has been found to be constitutively expressed at high levels throughout life in these organs [12]. In the lung, the basolateral membranes of alveolar type I epithelial cells and alveolar type II cells are where the expression is localized [15, 16]. However, the exact role or function of this high expression in the physiology of these cells remains poorly defined.

## 3. RAGE Isoforms

The membrane-bound form of RAGE consisting of three domains—extracellular domain, transmembrane domain, and cytosolic domain—is named full-length RAGE (fl-RAGE). Besides fl-RAGE, there are 19 naturally occurring splice variants for human RAGE that have been identified on mRNA and protein level [17]. These isoforms, including sRAGE1, sRAGE2, sRAGE3 [18], hRAGEsec [8], N-truncated and Secretory [19], RAGE_v4-RAGE_v13 [20], RageΔ, NtRAGEΔ, sRAGEΔ [21, 22], and Δ8-RAGE [23], are characterized by either N-terminal or C-terminal truncations. Later, Hudson and colleagues summarized all of these human RAGE isoforms and renamed them into RAGE_v1 to RAGE_v19 according to the Human Gene Nomenclature Committee [20]. As shown in Figure 1, both endogenous secretory RAGE (esRAGE or RAGE_v1) and cleaved RAGE (cRAGE) are soluble isoforms termed as soluble RAGE (sRAGE). These soluble isoforms have the same V-type and C-type regions (extracellular domain) as fl-RAGE but lacking the transmembrane and cytoplasmic domains [20, 24].

The two primary mechanisms that give rise to sRAGE are alternative mRNA splicing and proteolytic cleavage of fl-RAGE. Alternative splicing at exon 9 results in esRAGE, a C-truncated form of RAGE, while proteolytic cleavage of fl-RAGE at the cell surface gives rise to cRAGE, another soluble isoform of RAGE [19, 24]. sRAGE can act as a decoy receptor that intercepts the interaction of ligands with fl-RAGE because sRAGE, which occurs in the extracellular space, can bind RAGE ligands before they interact with fl-RAGE at the cell surface [3, 25, 26].

Human RAGE mRNA is subject to alternative splicing, resulting in a variety of splice variants. However, many of these splice sequences are likely to be degraded before they are expressed as proteins because these sequences are potential targets of the nonsense-mediated decay (NMD) pathway [20]. Some of the splice sequences might be able to undergo protein expression, but the expressed protein would be destroyed by premature degradation due to the absence of a signal sequence on exon 1 [20]. Studies have shown that alternative splicing in humans depends on the cell type or tissue. For example, in human lung and aortic smooth muscle cells, fl-RAGE mRNA is the most prevalent form, but in endothelial cells esRAGE mRNA instead of fl-RAGE mRNA is prevalent [20, 27].

Proteolytic cleavage that releases the extracellular domain of fl-RAGE as cRAGE is mediated by a membrane metalloproteinase called ADAM 10 [28, 29]. This isoform from proteolytic cleavage has been identified as the predominant species in serum. Hence, enhancing proteolytic cleavage will result in a rise in the level of sRAGE [28].

## 4. RAGE and Inflammation

Recent studies reporting increased expression of RAGE in a number of acute and chronic inflammatory diseases have suggested participation of RAGE and its downstream signalling pathways in perpetuating immune and inflammatory responses [30, 31]. This idea is further supported by various findings on the molecular mechanism of RAGE in contributing to the inflammatory response [31, 32]. 

First, RAGE has been found on numerous immune cells that play key roles in perpetuating the immune response. These cells include neutrophils, T and B lymphocytes, monocytes, macrophages, and also dendritic cells [33–35]. Second, many of the extracellular ligands that trigger RAGE signalling have been determined to be involved in acute and chronic immune responses [36, 37]. Third, RAGE expression has been found on endothelial cells, and this expressed RAGE can physically interact with the leukocyte *β*2 integrin Mac-1. The RAGE-Mac-1 interaction enables RAGE to function as an adhesion receptor for leukocytes [38–40]. Fourth, proinflammatory transcription factor nuclear factor kappa B (NF-*κ*B) and its downstream target genes are activated following engagement of RAGE. Among these NF-*κ*B regulated target genes, some of them are regulators of the adaptive and innate immune systems [41]. Interestingly, RAGE itself is also an NF-*κ*B regulated target gene, exhibiting a functional binding site for NF-*κ*B in its proximal promoter [42].

**Figure 2:**
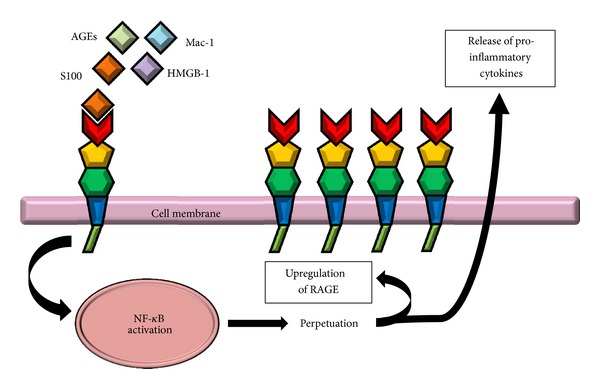
RAGE signalling resulting in sustained inflammation. Engagement of RAGE with various ligands including AGEs, HMGB1, S100 proteins, and *β*2-integrin Mac-1 leads to an intracellular signalling cascade ultimately resulting in perpetual activation of the transcription factor NF-*κ*B. Since the promoter region of RAGE contains two NF-*κ*B response elements, RAGE is upregulated where its ligands are present, associated with an increased secretion of proinflammatory cytokines, thereby promoting inflammatory cell recruitment.

Fifth, accumulation of RAGE ligands at sites of tissue injury and inflammation has been found to induce intracellular activation of NF-*κ*B [43]. RAGE-ligand interactions also lead to sustained NF-*κ*B signalling via *de novo *RelA (p65) mRNA synthesis because the *de novo* synthesis produces a constantly growing pool of proinflammatory transcriptionally active NF-*κ*B [44]. Since the signalling between RAGE and NF-*κ*B is interconnected, this ensures maintenance and amplification of the signal and thus sustains the cellular response (Figure 2). This sustained cellular response will in turn initiate chronic tissue alterations [41]. Sparvero et al. have extensively discussed and confirmed the role of RAGE and its ligands including HMGB1, S100 proteins, amyloid-beta peptide, and AGEs in inflammation [45]. The authors summarized that each of these ligands can distinctly activate RAGE signaling which in turn can perpetuate the immune and inflammatory responses via the activation of multiple intracellular signaling molecules such as NF-*κ*B, adhesion molecules, and MAP kinases [45].

Several immune cell types such as T lymphocytes, B lymphocytes, and macrophages have been found to express high levels of RAGE [46]. This high expression of RAGE has been closely linked to the activities of the immune cells as well as to inflammatory responses. The RAGE-Mac-1 interaction has been shown to mediate the adhesion of neutrophils and myelomonocytic cells to immobilized RAGE or RAGE-transfected cells [2, 47]. RAGE on T cells is associated with the differentiation of this cell type, as RAGE activation by its ligands has been determined to participate in the early events that eventually trigger the differentiation of Th1^+^ T cells [35]. RAGE expressed on T cells plays an essential role in the antigen-activated proliferative response. In a study on RAGE deficient T cells, production of the Th2 cytokines IL-4 and IL-5 was found to increase, while release of IL-2, IFN-*γ*, and Th1 was found to decrease. This finding suggests a contribution of RAGE activation in balancing Th1 and Th2 immunity [35].

In addition, several *in vivo* and *in vitro* findings have revealed that RAGE is involved in the recruitment of inflammatory cells by acting as a counter-receptor for leukocyte integrin. The ability of RAGE to become an endothelial cell adhesive receptor and to attract leukocytes enables RAGE to directly mediate the recruitment of leukocytes [36]. At the same time, RAGE-mediated cellular activation and upregulation of proinflammatory factors and adhesion molecules also indirectly increase the recruitment of inflammatory cells [36]. Also, Manfredi and colleagues have identified that RAGE expression on maturing dendritic cells is essential in order for this cell type to migrate to draining lymph nodes [48].

Although RAGE has been shown to be implicated in both acute and chronic immune responses, the exact regulatory mechanism of this receptor in inducing acute versus chronic inflammation remains unclear. To date, two possible strategies have been suggested to answer this question. The first hypothesis, proposed by Herold and colleagues, has linked the oligomerization status of RAGE ligands to RAGE-mediated chronic versus acute inflammatory responses [32]. In their study, it was proposed that oligomeric ligands have higher affinity for RAGE and thus are capable of inducing persistent signalling which leads to chronic inflammation. In contrast, monomeric ligands with lower affinity to RAGE can only elicit an acute response [32]. Notably, several lines of evidence from a recent study comparing the S100B tetramer with its dimeric counterpart further support the hypothesis mentioned above. The S100B tetramer exhibited higher affinity in binding sRAGE *in vitro* and improved cell survival more effectively compared with the dimeric form [11].

On the other hand, based on the same RAGE-ligand affinity concept, the second hypothesis suggests that the origin of the ligands may be a critical determinant in triggering acute versus chronic inflammation [49, 50]. It has been proposed that the patterns of the endogenous ligands have higher affinity for RAGE. Hence, the receptor induces chronic inflammation when exposed to the persistent endogenous danger signals [49, 50]. In contrast, the patterns of the exogenous ligands have lower affinity to RAGE and thus trigger acute inflammation [49, 50]. Tian and colleagues have suggested that RAGE may work together with toll-like receptors to elicit acute responses and rapid clearance in response to exogenous ligands like invading pathogens [50]. However, more experimental lines of evidence and systematic kinetic studies of RAGE-ligand interactions are required to resolve these two hypotheses.

## 5. RAGE Involvement in Inflammatory Diseases

The multiligand nature of RAGE provides this receptor the ability to engage a broad range of proinflammatory ligands. Binding of these ligands to RAGE leads to the recruitment of multiple intracellular signalling molecules, including the transcription factor NF-*κ*B, MAP kinases, and adhesion molecules, and eventually activates pathways responsible for acute and chronic inflammation [45]. Due to the role of RAGE as a central player in perpetuating and amplifying inflammatory responses, more and more studies connect this receptor to a number of pathological settings.

### 5.1. RAGE and Atherosclerosis

Atherosclerosis is a disease of the arteries characterized by plaque buildup on the inner wall and has been described as a chronic inflammatory disease for years [51]. RAGE has been linked with this inflammatory disease due to its presence on the surface of a wide variety of cells implicated in atherogenesis and progression of atherosclerosis, such as endothelial cells, lymphocytes, monocyte-derived macrophages, and vascular smooth muscle cells [52]. The involvement of several RAGE ligands including AGEs, amphoterin, and S100 proteins in the atherosclerotic process further affirm the relevance of RAGE involvement in atherosclerosis [5, 6, 53]. At sites of vascular injury, engagement of RAGE by its ligands would create an oxidant milieu by inducing increased generation of intracellular reactive oxygen species (ROS) [52, 54] via the NAD(P)H-oxidase system [55]. These accumulated ROS would activate the redox-sensitive transcription factor (NF-*κ*B) which in turn results in transcriptional activation of a variety of genes that are highly relevant for inflammation and atherosclerosis, such as tumour necrosis factors (TNF-*α*, TNF-*β*), interleukins (IL-1, IL-6, IL-8), interferon (IFN-*γ*), and cell adhesion molecules [56–58]. Hence, this oxidative stress can be said to be the key mediator of atherogenic changes in the vasculature as well as the first and most important pathological consequence of RAGE-ligand interaction.

Endothelial dysfunction has been proposed to be a priming event in the initiation of atherosclerosis [59] wherein the endothelium becomes faulty and vulnerable to the invasion of inflammatory cells and lipids—a key step in atherosclerotic plaque formation [60]. During the initiation and progression of atherosclerosis, RAGE is evidently expressed in endothelial cells, ready to transduce the impact of its ligands, which can lead to endothelial dysfunction [61]. When AGEs bind to RAGE expressed on endothelium, activation of RAGE changes the antithrombotic phenotype of the endothelium by reducing thrombomodulin activity and concomitantly inducing expression of tissue factor. This alters the dynamic endothelial properties, transforming the anticoagulant endogenous surface to a surface that is procoagulant to the flowing blood [62, 63]. In addition, the interaction of RAGE and AGEs enhances the expression of adhesion molecules including E-selectin, intercellular adhesion molecule-1 (ICAM-1), and vascular adhesion molecule-1 (VCAM-1) via NF-*κ*B activation [56]. A number of studies have demonstrated induction of VCAM-1 expression in a RAGE-dependent manner when endothelial cells are exposed to AGEs and also other RAGE ligands like S100A12 (EN-RAGE) or S100B [6, 56]. High expression of adhesion molecules in endothelial cells may promote adhesive interactions of circulating monocytes with the endothelial surface, and this can eventually result in transendothelial migration [64].

Besides endothelial cells, mononuclear phagocytes (monocytes/macrophages) are another highly relevant cell type that has been shown to express RAGE in the context of atherosclerosis. Mononuclear infiltration into the subendothelial space along a chemotactic gradient after adhesion of monocytes to endothelial cells has been recognized as characteristic of the development of atherosclerosis [65]. After the transmigration, these monocytes differentiate into intimal macrophages that in turn transform into foam cells and accumulate in the blood vessel wall, speeding up fatty streak formation [65]. Engagement of AGEs to RAGE on mononuclear phagocytes has been reported to give rise to this phenotype of activated macrophages that is manifested by the induction of some proinflammatory cytokines (IL-1*β* and TNF-*α*), platelet-derived growth factor, and also insulin-like growth factor-1 [66, 67]. Interestingly, these mediators all play pivotal roles in the pathogenesis of atherosclerosis [51]. Similar findings were revealed when Hofmann and colleagues used S100A12 to bind to RAGE on cultured murine macrophages called Bv2 cells; they found that IL-1*β* and TNF-*α* were induced in an NF-*κ*B-dependent manner [6]. Binding of soluble RAGE ligands such as AGEs and S100A12 to RAGE-bearing mononuclear phagocytes prompts chemotaxis and subsequently results in mononuclear infiltration through an intact endothelial monolayer [13, 68].

AGEs have been found to be closely associated with increased expression of various oxidized LDL (oxLDL) receptors including macrophage scavenger receptor, CD36 receptor, and lectin-like oxLDL receptor 1 on human monocyte-derived macrophages [69]. The increased numbers of oxLDL receptors on macrophage membranes consequently enhance the uptake of modified LDL. Gene expression of these oxLDL receptors is reported to reach its peak in enhanced foam cell transformation [70]. Furthermore, AGEs-RAGE binding reduces the expression of ATP-binding cassette transporter G1 and decreases the efflux of cholesterol to high-density lipoprotein (HDL) [70]. Hence, the AGEs-RAGE interaction has been proposed to contribute to foam cell formation by increasing oxLDL receptors as well as decreasing cholesterol efflux to HDL.

Further development of fatty streak lesions into advanced lesions that can cause thromboembolic events is usually associated with smooth muscle cell accumulation, necrotic core formation, lipid accumulation, and also the formation of a fibrous cap [71, 72]. In smooth muscle cells, binding of AGEs to RAGE triggers an increase in chemotactic migration and cellular proliferative activity as well as production of fibronectin [71, 72]. Several RAGE-mediated signalling pathways such as SRC kinase, MAP kinases, JAK-STAT, and NF-*κ*B have been shown to be implicated in cellular migration and proliferation [73–76]. AGEs can upregulate a key regulator named transforming growth factor-*β* (TGF-*β*) in order to mediate extracellular matrix generation by smooth muscle cells [77]. In addition to AGEs, exposure of smooth muscle cells to amphoterin can also induce cellular proliferation, migration, and the secretion of more amphoterin [78, 79]. Another RAGE ligand, S100B, has also been found to contribute to the atherogenic process in a RAGE-dependent manner via increased activation of Src kinase, tyrosine phosphorylation of caveolin-1, MAPKs, NF-*κ*B, and STAT3, as well as induction of smooth muscle cell migration and superoxide production [73]. Taken together, these findings gathered so far unequivocally underscore the fundamental roles of the RAGE-ligand axis in the pathogenesis of vascular dysfunction and ultimately atherosclerosis.

### 5.2. RAGE and Alzheimer's Disease

Alzheimer's disease (AD) is a progressive neurodegenerative disorder and the most common cause of dementia in the elderly, hallmarked by a progressive decline in cognitive functions [80]. AD pathology is characterized by the presence of senile plaques and neurofibrillary tangles as well as severe gliosis in both the cerebral cortex and hippocampus [81]. Also, increased oxidative stress, amplified inflammatory responses, and dysregulation of calcium homeostasis have been observed in the AD brain [82]. During the development and progression of AD, expression of RAGE is found to be upregulated in cells surrounding the senile plaques such as microglia, neurons, and endothelial cells [83, 84]. The exact role of RAGE in the pathogenesis of AD is not yet clearly known, but activation of RAGE by ligands that are closely linked to AD, including beta amyloid peptide (A*β*), AGEs, and S100 proteins, appears to trigger several signal transduction cascades leading to neuronal loss.

Binding of AGEs and A*β* to RAGE has been reported to stimulate activation of transcription factor NF-*κ*B which in turn induces the release of various cytokines such as IL-1, IL-6, TNF-*α*, endothelin-1, and tissue factor [41, 44, 85]. Activation of NF-*κ*B was found to be involved in neuronal plasticity and the cellular response to neurodegeneration [86], while prolonged activation of RAGE can lead to cellular dysfunction [41]. Interestingly, activation of NF-*κ*B can create a positive loop to amplify the cellular response to external stress by upregulating the expression of RAGE [41]. Moreover, binding of AGEs to RAGE stimulates the generation of reactive oxygen species (ROS) by activating NADPH oxidase (NOX), and the ROS produced have been implicated in the early toxic events that result in progression of AD [87, 88]. 

Increased expression of RAGE and two of its ligands, A*β* and AGEs, have been identified in AD hippocampus, particularly in dentate gyrus neurons and CA3 pyramidal neurons. This finding corresponds with the short-term memory loss in AD patients caused by neuronal dysfunction in the hippocampus [89]. Recently, two studies have revealed that activation of RAGE by either AGEs or A*β* can enhance the expression of BACE 1, a key enzyme in promoting the production of A*β* in the brain [87, 90]. Besides the hippocampus, the entorhinal cortex, which is also an important brain area in memory processing, has been found to be affected early in AD. RAGE has been demonstrated to be implicated in A*β*-dependent impaired synaptic transmission in the entorhinal cortex, as confirmed by the inhibitory effect of an anti-RAGE antibody [91]. Furthermore, another study has shown that RAGE contributes to inhibition of synaptic plasticity induced by A*β* in intracortical circuits of the visual cortex [92]. Both A*β* and AGEs have been reported to decrease mitochondrial activity of neuronal cells and induce neurodegeneration via mitochondrial dysfunction [93, 94]. In a RAGE-dependent manner, the uptake of A*β* and A*β* targeting mitochondria in cortical neurons causes the activity of cytochrome c oxidase (COX IV), a key mitochondrial respiratory enzyme, to decrease [95]. The role of RAGE in A*β*-mediated neurodegeneration was further confirmed when treatment with an anti-RAGE antibody was seen to diminish A*β* targeting to mitochondria and also the subsequent mitochondrial damage [95].

Activated astrocytes and microglia are common in AD brains with chronic inflammation, and the presence of these activated cells can result in chronic oxidative stress [96]. Notably, oxidative stress stimulates the formation of AGEs [96]. These AGEs will activate RAGE, which in turn triggers more oxidative stress, thus creating a positive feedback loop [30, 85]. Moreover, RAGE activation by A*β* upregulates macrophage colony stimulating factor (M-CSF) expression in neuronal cells, which will then enhance proliferation and release of proinflammatory cytokines in microglia [4]. Fang and colleagues found enhanced induction of proinflammatory cytokines (IL-1*β* and TNF-*α*), increased infiltration by microglia and astrocytes, and increased A*β* plaque load in their study on mutant amyloid precursor protein (mAPP) transgenic mice [97]. This evidence indicates that RAGE plays a key role in facilitating activated microglial effects that will ultimately impair neuronal function [97]. 

*In vivo* studies show the interaction of RAGE and A*β* at the luminal membrane of the blood-brain barrier (BBB). This finding proposes that RAGE can be a transporter protein at the BBB, facilitating the transport of circulating A*β* across the BBB [98]. This is supported by another study in which RAGE mediated the entry of A*β*1-40 and A*β*1-42 into the hippocampus and cortex across the BBB [99]. The role of RAGE as a BBB transporter protein was further confirmed in another study when A*β* transport was inhibited in RAGE null mice as well as in mice treated with anti-RAGE antibodies [100]. Also, in the same study, Deane and colleagues demonstrated that the A*β*-RAGE interaction at the BBB not only results in neurovascular stress and expression of proinflammatory cytokines (TNF-*α* and IL-6) but also leads to decreased cerebral blood flow by enhancing the secretion of endothelin-1 to induce vasoconstriction [100]. Transmigration of blood-derived or bone-marrow-derived monocytes along with A*β* depositions have been found in the diseased AD brain [101]. This A*β*-induced monocyte infiltration has been shown to be inhibited by blockade of RAGE with anti-RAGE antibodies [102]. 

Taken together, all the evidence obtained from previous studies supports the critical involvement of RAGE in AD progression.

### 5.3. RAGE and Arthritis

Arthritis is a form of joint disorder frequently accompanied by arthralgia and stiffness of the affected joint. Among the over 100 types of arthritis identified, osteoarthritis (OA) and rheumatoid arthritis (RA) are the two most common types [103, 104]. Herein, these two types of arthritis will be further discussed. OA is a “wear and tear” degenerative joint disease featuring degradation of articular cartilage and subchondral bone accompanied by secondary low-grade inflammation of the synovial tissue [103]. RA, on the other hand, is a chronic autoimmune disease that is hallmarked by prolonged inflammation of synovial tissue, bone erosion, and cartilage degradation [104]. Despite the differences in the underlying pathophysiology, both OA and RA eventually result in joint dysfunction and disability.

RAGE has been detected in synovial tissue, macrophages, T cells, and some B cells in the affected joints of both OA and RA patients [105]. As all these cells are implicated in the development of synovial inflammation in RA and OA, this suggests a role for RAGE in the pathogenesis of both joint diseases, especially RA [105]. Interestingly, various studies have reported not only the presence but also upregulation of RAGE in focal degenerated cartilage of OA [106], as well as in synovial tissue macrophages and infiltrating lymphocytes of RA [107]. In parallel with the upregulated receptor, several ligands of RAGE including AGEs, S100 calgranulins, and HMGB-1 are found to accumulate at sites of OA and RA [108–111]. 

The presence of RAGE and its ligand AGEs has been confirmed in the synovial lining, sublining, and endothelium of OA patients [105, 108], and increased concentrations of the ligands were found in serum, synovial fluid, and urine obtained from OA patients [108, 112]. Accumulation of AGEs has been found in OA cartilage collagen [110], and these accumulated ligands are capable of altering the mechanical properties and metabolism of cartilage via RAGE signalling [113, 114]. Studies revealed that an increased level of AGEs in cartilage significantly increases cartilage stiffness, which may result in failure of the cartilage to resist damage [113, 114]. Moreover, it has been found that accumulation of AGEs impairs the synthesis of matrix molecules by articular chondrocytes, resulting in decreased collagen turnover and decline in proteoglycan synthesis rate [115, 116]. So, activation of RAGE on chondrocytes by accumulated AGEs can cause a stiffer matrix and impair the synthetic capacity of the cells. These may in turn diminish the capacity of articular chondrocytes to maintain matrix integrity after injury and thus increase susceptibility to OA.

Besides altering chondrocyte activity, activation of RAGE was also reported to affect synoviocyte activity and thus contribute to the pathogenesis of OA. There was a study proposing that the fibroblast-like synoviocyte (FLS) appeared to play an outstanding role in the pathogenesis of OA [117]. Another study showed that inflammation and cartilage degradation were inhibited in mice immunized and challenged with collagen type II (CII) when the mice were treated with sRAGE [118]. Also, Steenvoorden and colleagues found that activation of RAGE on chondrocytes and synoviocytes by AGEs could substantially enhance production of MMP-1, invasiveness, and proteoglycan release by the cells [106]. Hence, elevated AGE levels can alter the activities of both chondrocytes and synoviocytes in OA joints and increase cartilage degradation by inducing catabolic pathways via RAGE activation. This may be one of the molecular mechanisms that cause tissue degradation in OA to continue. In addition to AGEs, increased levels of HMGB-1 and several S100 calgranulins such as S100A11, S100A4, and S100B have also been reported in osteoarthritic cartilage [109, 111, 119]. Binding of S100A11 to RAGE was found to trigger chondrocyte hypertrophy [119]. On the other hand, HMGB-1, S100A4, and S100B were demonstrated to stimulate articular chondrocytes to produce matrix metalloproteinase 13 (MMP-13), indicating increased cartilage degradation [109, 111].

Similar to OA, increased levels of RAGE ligands including AGEs (pentosidine and N-carboxymethyllysine), S100 calgranulins (S100A4, S100A8, and S100A9), and HMGB-1 have been reported in RA patients [108, 111, 112, 120, 121]. Previous studies found that the levels of pentosidine and S100A12 correlate with disease activity in RA [122, 123], while S100A4 is reported to induce MMP-13 production just like in OA [111]. In addition, HMGB-1 has been shown to induce synovial fluid macrophages to release TNF-*α*, IL-1*β*, and IL-6 by RAGE signalling [124, 125]. Steenvoorden and colleagues have demonstrated that either HMGB-1 or glycated albumin increases the invasiveness of synoviocytes in a RAGE-dependent manner, as confirmed by the inhibitory effect of anti-RAGE antibody [126].

All these findings indicate that RAGE does play an important role in the development and progression of RA. Although there are differences between the pathologies of OA and RA, the data gathered so far raise the intriguing hypothesis that RAGE activation by elevated levels of its ligands contributes to the cartilage degradation seen in both OA and RA.

### 5.4. RAGE and Pulmonary Disease

Most tissues display a relatively low expression of RAGE in their normal physiological state. However, lung has been identified as an exception, as RAGE is constitutively expressed at a high basal level in pulmonary tissue, localized primarily in alveolar type I (ATI) pneumocytes [127]. Alterations in RAGE levels and RAGE-ligand interaction have been suggested to play a relevant role in the pathogenesis of several pulmonary diseases. Acute lung injury (ALI) and acute respiratory distress syndrome (ARDS), a more severe manifestation, are syndromes of acute respiratory failure, hallmarked by hypoxemia, disruption of alveolar fluid clearance (AFC), deterioration of the alveolar-capillary barrier, and, most importantly, damage to ATI pneumocytes [128, 129].

Increased levels of RAGE were demonstrated in bronchoalveolar lavage fluid (BALF) in various direct models of lung injury induced separately by intratracheal instillation of hydrochloric acid, lipopolysaccharide (LPS), or *Escherichia coli* as well as exposure to hyperoxia [130]. Notably, RAGE deletion in mice was reported to exhibit protective effects against hyperoxia-induced mortality and diminish characteristics of hyperoxia-induced ALI [131]. RAGE null mice exposed to hyperoxia survived significantly longer and showed a marked reduction in total BALF cells, total protein leakage, and secretion of proinflammatory cytokines in BALF [131]. Also, increased RAGE levels in BALF, together with inflammation and damage, were found in mice after intratracheal insufflation of IL-1*α* and IFN-*γ* cytokines [132].

In a different study, sRAGE levels in BALF were reported to elevate in response to LPS challenge [133]. Further, sRAGE which acted as a decoy receptor and blocked RAGE signalling to alleviate the inflammatory events was administered into the mice after LPS instillation. These sRAGE-treated mice were shown to display a significant reduction in neutrophil infiltration, lung permeability index, and NF-*κ*B activity, as well as production of several proinflammatory cytokines including TNF-*α* and macrophage inflammatory protein (MIP-1*α* and MIP-1*β*) in BALF [133]. RAGE has been found to be a promising marker of ATI cell injury [134]. Due to the role of ATI cells in epithelial integrity and alveolar fluid clearance, RAGE is proposed to be a biomarker for the severity of ALI/ARDS clinical outcomes [134]. Consistent with these findings, Calfee and colleagues showed that higher baseline plasma RAGE levels are significantly correlated with increased severity of lung injury [135].

Besides the receptor, a few ligands of RAGE have been linked to lung injury. Amplified pulmonary S100A12 expression and higher BALF concentrations of S100A12 protein have been demonstrated in ARDS patients [136]. Instillation of another RAGE ligand, HMGB-1, has been found to trigger neutrophil accumulation, development of lung oedema, and elevated pulmonary levels of cytokines such as IL-1*β*, TNF-*α*, and MIP-2 [137, 138]. As the ligands mentioned above primarily act via RAGE signalling, these findings indicate the role of RAGE in inducing acute inflammatory lung injury.

Furthermore, RAGE has been associated with asthma which is a chronic inflammatory disease of the airway. The hallmarks of this lung disease include airway obstruction, increased airway responsiveness, and airway inflammation which involve various cell types and inflammatory mediators [139]. In asthma, airway inflammatory response usually involves airway neutrophilia which is characterized by ongoing neutrophil influx, uncontrolled neutrophil activation, and impaired neutrophil clearance [140]. Neutrophilic airway inflammation has been closely related to severe asthma [141, 142] and is found to be implicated in the development of persistent airflow limitation which is one of the hallmarks of severe asthma [143]. These findings are further supported by another study reporting that patients with severe or refractory asthma frequently exhibit neutrophilic airway inflammation [144].

Watanabe and colleagues have proposed that one of the important RAGE ligands, HMGB1 protein, may enhance neutrophilic inflammation and may play a role in neutrophilic asthma [144]. In the same study, higher levels of HMGB1 protein and esRAGE have been observed in asthmatic sputum, but only increased levels of HMGB1 were found to be associated with severity of asthma [144]. Another recent study demonstrated positive correlations between the levels of HMGB1 or RAGE and the percentage of neutrophils in asthma patients [145]. This study also showed that both HMGBl and RAGE expressions in the asthma patients were reduced after receiving treatment. Hence, the authors have suggested that both HMGB1 and RAGE may play a role in inflammatory process and pathogenesis of asthma [145]. Additionally, patients with neutrophilic asthma were reported to express significantly lower systemic levels of sRAGE, indicating a positive correlation between reduced sRAGE and neutrophilic airway inflammation in asthma [146].

In rodent model of asthma induced by either house dust mite (HDM) or ovalbumin, RAGE deletion has been demonstrated to protect the mice by eliminating airway remodeling, eosinophilic inflammation, and airway hypersensitivity irrespective of the type of allergens involved [147]. The same study also showed that inhibition of RAGE in wild type mice can significantly reduce inflammation in asthma [147]. All of these results suggest that RAGE-ligand axis plays a role in neutrophilic airway inflammation as well as in the pathogenesis of asthma.

In addition to ALI/ARDS and asthma, a growing body of evidence supports the involvement of RAGE in pulmonary fibrosis. Significant reductions in the levels of both sRAGE and membrane RAGE have been observed in a series of animal models of pulmonary fibrosis, in which administrations of bleomycin, asbestos or silica are used to induce lung injury [148–150]. This deleterious effect on the expression of RAGE has also been found in ATI cells extracted from lung slices of rats exposed to CdCl2 and TGF-*β*1 [151]. Similarly, reduced RAGE concentrations have been shown in lung homogenates and BALF of idiopathic pulmonary fibrosis (IPF) patients [148, 152]. A study on RAGE null mice has revealed that these mice spontaneously develop fibrosis-like alterations in lungs and develop more severe fibrosis compared to wild-type controls when subjected to a model of pulmonary fibrosis induced by asbestos [148]. All these findings indicate that loss of RAGE may contribute to the pathogenesis of pulmonary fibrosis.

However, conflicting results from studies on mouse models of pulmonary fibrosis and human IPF tissues have been found. An investigation by He and colleagues showed that RAGE null mice were largely protected from bleomycin-induced lung injury, accompanied by decreased levels of potent RAGE-inducible profibrotic cytokines TGF-*β*1 and PDGF in BALF, and improved survival [153]. Moreover, overexpression of RAGE was observed in reactive pneumocytes, bronchiolar metaplastic epithelium, and endothelium in IPF lungs. This study also found that extensive RAGE reactivity was more evident in fibroblastic foci where inflammatory cells aggregate [154].

The exact role of RAGE in the pathogenesis of pulmonary diseases remains unclear due to the conflicting results from different current studies. However, RAGE undoubtedly plays an important role in both physiological and pathological conditions of the lung.

### 5.5. RAGE and Sepsis

Sepsis is a heterogeneous clinical syndrome defined as a systemic inflammatory response to infection. It is characterized by a wide range of systemic and organ function aberrations which subsequently result in tissue injury and organ failure [155]. RAGE has been proposed to be involved in the pathogenesis of sepsis due to its role in transmitting signals from pathogen substrates to activate cells during the onset and perpetuation of inflammation. Studies have demonstrated that ligands of RAGE, S100 calgranulins, and HMGB-1 are elevated in septic patients, and this further supports the role of RAGE in the pathogenesis of sepsis [156–158]. Although the exact role of RAGE in sepsis still remains a puzzle, more and more evidence from animal studies has been documented. 

In a model of cecal ligation and puncture (CLP)-induced sepsis, a significant improvement in survival and higher arterial oxygenation were observed in RAGE null mice as compared with wild-type controls [159]. Decreased activation of NF-*κ*B was also noted in RAGE null mice, thus suggesting that activation of NF-*κ*B is probably the mechanism underlying the protective effects of RAGE deletion [159]. This model of CLP-induced sepsis was also used in another study to test the effect of RAGE blockade with anti-RAGE antibody. The study showed that administration of a rat anti-murine RAGE monoclonal antibody significantly increased the survival rate in mice undergoing CLP [160]. This significant survival benefit was even observed in mice receiving the anti-RAGE antibody if it was delayed for 24 hours [160]. Noteworthy, the same study tested the effects of RAGE blockade and RAGE deletion in a model of systemic listeriosis using *Listeria monocytogenes* infection and found the same protective effects as in the CLP model of polymicrobial sepsis [160]. Furthermore, Van Zoelen and colleagues have demonstrated an improved survival in RAGE null mice and an improved killing capacity of* Streptococcus pneumonia* in RAGE-deficient macrophages *in vitro* [161]. The authors suggest that the improved host defence may be the underlying cause for the improved survival in RAGE null mice during pneumococcal pneumonia [161].

All the evidence from animal models of sepsis underlines that RAGE is implicated, at least in part, in the pathogenesis of sepsis. However, further research is warranted to confirm the exact role of RAGE, especially the role of RAGE in critical organ derangements during sepsis pathogenesis.

## 6. Concluding Remarks/Perspectives

From all the evidence presented in this review, it is obvious that the RAGE-ligands axis is closely linked to a broad range of diseases, all of which appear to exhibit upregulation and accumulation of one or more types of RAGE ligands. RAGE, expressed in many different cell types, interacts with a number of its ligands to model a complicated biochemical axis linking complex which in turn perpetuates and amplifies inflammatory responses and leads to the pathogenesis of various inflammatory-related diseases.

There is a growing body of evidence connecting RAGE-ligand axis to a number of pathological settings such as cardiovascular disease, neurodegeneration, diabetes mellitus, and immune/inflammatory diseases [162]. This has evoked a focus of attention to the potential of RAGE as a target of therapeutic intervention. To investigate the effects of RAGE blockade in pathological conditions, many studies have used soluble form of RAGE (sRAGE) which can antagonize RAGE-ligand interaction to competitively inhibit the activation of RAGE signalling [46, 163–165]. Evidence from these studies has shown that RAGE blockade protected the experimental animals against various disease challenges. The potential impact of RAGE deletion has been further studied in homozygous RAGE deficient mice to investigate the therapeutics possibilities of RAGE [131, 160, 161, 166]. These studies have reported that genetic deletion of RAGE also provided protection and improved survival in RAGE null mice during clinical settings.

Based on the impressive results obtained from animal studies, RAGE blockade or RAGE deletion may be proved beneficial in clinical settings [46, 131, 160, 161, 163–166]. Hence, there is no doubt that RAGE is a potential pharmacotherapeutic target. However, there are at least two critical issues that remain to be resolved. The first is the advantages and disadvantages of such a RAGE-targeting therapy in humans. The second is the effect of prolonged RAGE blockade in human subjects. This is important because RAGE plays vital roles in normal physiology. Future investigations are definitely required to develop a greater understanding of the impact of RAGE blockage before a promising RAGE-directed therapeutic strategy can be established.
